# Gut dysbiosis in autoimmune diseases: Association with mortality

**DOI:** 10.3389/fcimb.2023.1157918

**Published:** 2023-03-31

**Authors:** Sung-Ho Chang, Youngnim Choi

**Affiliations:** Department of Immunology and Molecular Microbiology, School of Dentistry and Dental Research Institute, Seoul National University, Seoul, Republic of Korea

**Keywords:** autoimmunity, gut dysbiosis, Sjögren’s syndrome, rheumatoid arthritis, systemic lupus erythematosus, multiple sclerosis

## Abstract

To better understand the impact of gut dysbiosis on four autoimmune diseases [Sjögren’s syndrome (SS), systemic lupus erythematosus (SLE), rheumatoid arthritis (RA), and multiple sclerosis (MS)], this review investigated the altered gut bacteria in each disease and the shared ones among the four diseases. The enriched gut bacteria shared by three of the four autoimmune diseases were *Streptococcus*, *Prevotella*, and *Eggerthella*, which are associated with autoantibody production or activation of Th17 cells in immune-related diseases. On the other hand, *Faecalibacterium* comprises depleted gut bacteria shared by patients with SLE, MS, and SS, which is associated with various anti-inflammatory activities. The indexes of gut dysbiosis, defined as the number of altered gut bacterial taxa divided by the number of studies in SLE, MS, RA, and SS, were 1.7, 1.8, 0.7, and 1.3, respectively. Interestingly, these values presented a positive correlation trend with the standardized mortality rates —2.66, 2.89, 1.54, and 1.41, respectively. In addition, shared altered gut bacteria among the autoimmune diseases may correlate with the prevalence of polyautoimmunity in patients with SLE, SS, RA, and MS, that is, 41 percent, 32.6 percent, 14 percent, and 1–16.6 percent, respectively. Overall, this review suggests that gut dysbiosis in autoimmune diseases may be closely related to the failure of the gut immune system to maintain homeostasis.

## 1 Introduction

The etiology of autoimmune diseases is complex involving both genetic and environmental factors. Genetic risk factors for autoimmune diseases are composed of HLA and non-HLA genes expressed at different levels depending on the disease (Greiling et al., 2018; van der Meulen et al., 2019; Frazzei et al., 2022). On the other hand, environmental factors include smoking, lifestyle disorders, reduced sun exposure, and chronic stress (Frazzei et al., 2022). However, the scope of these factors to explain the cause of the rapid increase in autoimmune diseases over the decades is insufficient (Chen et al., 2017; Dinse et al., 2022; Frazzei et al., 2022). Recently, gut dysbiosis has attracted great attention as a risk factor for autoimmune diseases. However, it is unclear whether gut dysbiosis is a result or a cause of an autoimmune disease (Jubair et al., 2018). Autoimmune diseases such as primary Sjögren’s syndrome (SS), systemic lupus erythematosus (SLE), rheumatoid arthritis (RA), and multiple sclerosis (MS) have been linked to gut dysbiosis (Zhang et al., 2015; Chen et al., 2016a; Nikitakis et al., 2017; Luo et al., 2018; Bellando-Randone et al., 2021). The causes of gut dysbiosis include depletion of the mucus layer, rapid dietary changes, use of antibiotics, infection and inflammation, and gastrointestinal surgery (Van de Wiele et al., 2016). Chen et al. proposed the following five mechanisms by which gut dysbiosis contributes to autoimmune diseases: 1) dysregulation of TLR in antigen presenting cells (APCs) and imbalance of Treg/Th17 ratio; 2) generation of new autoantigens due to the modification of host proteins induced by microbial enzymes; 3) microbial components similar to self-peptides, activating autoreactive B and T cells; 4) induction of immunopathology through the transport of microbial components or metabolites throughout the host; and 5) autoantibody generation against curli-DNA composites (Chen et al., 2017). In this review, the altered gut bacteria in SLE, MS, RA, and SS were investigated to better understand the impact of gut dysbiosis on autoimmune diseases. First, we investigated whether there are common taxa in different studies of gut dysbiosis for each disease. Second, we investigated whether the four autoimmune diseases share altered gut bacteria and whether there are altered gut bacteria unique to each autoimmune disease. Third, the altered gut bacteria’s functions or mechanisms of action in immune-related diseases were investigated. Fourth, we explored whether the shared, altered gut bacteria are related to polyautoimmunity. Finally, we examined whether the degree of gut dysbiosis is related to the disease’s standardized mortality rate (SMR).

## 2 Gut dysbiosis in autoimmune diseases

Altered gut bacteria refer to the taxa whose enrichment or depletion in the gut bacteriota has been cross-validated by at least two studies with statistical significance (p < 0.05, q < 0.1, or false discovery rate < 0.1). The taxonomic range of altered gut bacteria investigated in the four autoimmune diseases was at the family, genus, or species levels. Most altered bacteria were cross-validated at the genus or species levels because some papers only presented them at the genus or species levels.

### 2.1 Altered gut bacteria in SLE

SLE is a prototypical autoimmune disease associated with loss of self-tolerance of the immune system, abnormal antibody response to cytoplasmic antigens, persistent autoantibody production, and subsequent systemic inflammation (Chen et al., 2021). Its clinical signs include multiple symptoms, such as skin rash, glomerulonephritis, neurological disorders, and severe vasculitis, suggesting that the pathogenesis of SLE may be complex (Guo et al., 2020). A total of 22 altered gut bacterial genera/families were identified from the review of 13 papers published since 2014 (**Table 1**). While *Alistipes*, *Bacilli*, *Bacteroides*, *Clostridium*, *Eggerthella*, *Escherichia*, *Klebsiella*, *Lactobacillus*, *Prevotella*, *Ruminococcus*, and *Streptococcus* are enriched, *Bacteroides*, *Dialister*, *Faecalibacterium*, *Odoribacter*, *Roseburia*, and *Ruminococcus* are depleted in the gut of SLE patients. Interestingly, *Bacteroides* and *Ruminococcus* were reported to be enriched or depleted depending on studies, but at the species level, different species were enriched or depleted with the exception of *B. uniformis*, which was reported to be enriched or depleted in different studies (**Table 1**). Even in the same species, *Bacteroides fragilis* is classified into polysaccharide A-producing beneficial bacterium or enterotoxigenic bacterium, depending on strains (Nagao-Kitamoto and Kamada, 2017). Thus, *B. fragilis* enrichment in patients with SLE might be associated with enterotoxigenic strains. Among the 22 altered gut bacterial genera, *Bacteroides*, *Escherichia*, *Ruminococcus*, and *Streptococcus* have known functions associated with the induction of inflammatory response or autoimmunity in immune-related diseases (Vatanen et al., 2016; Cunningham, 2019; Henke et al., 2019; Qiu et al., 2019), whereas *Faecalibacterium* and *Ruminococcus*_2 have anti-inflammatory mechanisms of action (Houtman et al., 2022; Matsuoka et al., 2022). More details about this phenomenon have been described in Section 2.6.

**Table 1:** Commonly altered gut bacteria in gut dysbiosis of patients with autoimmune diseases.

Diseases (Ref count/Altered gut bacteria count)	Enrichment	Ref	Depletion	Ref
SLE (13/22)	*Alistipes* *Bacilli* *Bacteroides (fragilis, ovatus, uniformis, vulgatus)* *Clostridium (innocuum, leptum)* *Eggerthella (lenta)* *Enterobacteriaceae* *Escherichia (coli, shigella)* *Klebsiella* *Lactobacillaceae* *Lactobacillus (mucosae*, *salivarius)* *Prevotella* *Ruminococcus (gnavus*, *torques)* *Streptococcaceae* *Streptococcus (anginosus*, *mutans, oligofermentans, parasanguinis)*	(Guo et al., 2020; Chen et al., 2021) (Bellocchi et al., 2019; Li et al., 2019) (van der Meulen et al., 2019; Guo et al., 2020; Chen et al., 2021; Zhang et al., 2021) (Chen et al., 2021; Wen et al., 2021) (He et al., 2016; Chen et al., 2021) (Bellocchi et al., 2019; Wen et al., 2021) (He et al., 2020; Chen et al., 2021; Wen et al., 2021) (He et al., 2016; Wen et al., 2021; Zhang et al., 2021) (Bellocchi et al., 2019; Li et al., 2019) (Bellocchi et al., 2019; Li et al., 2019; Chen et al., 2021) (He et al., 2016; Guo et al., 2020) (Azzouz et al., 2019; Chen et al., 2021; Wen et al., 2021; Zhang et al., 2021) (Li et al., 2019; Wen et al., 2021; Zhang et al., 2021) (Bellocchi et al., 2019; Li et al., 2019; Chen et al., 2021; Wen et al., 2021)	*Bacteroides (cellulosilyticus, eggerthii, intestinalis, plebeius, salyersiae, uniformis)* *Dialister* *Faecalibacterium (prausnitzii)* *Lachnospiraceae* *Odoribacter* *Roseburia* *Ruminococcaceae* *Ruminococcus (2, callidus, lactaris, obeum)* *Firmicutes/Bacteroidetes ratio	(Azzouz et al., 2019; Chen et al., 2021) (He et al., 2016; Wen et al., 2021) (Li et al., 2019; Chen et al., 2021; Wen et al., 2021; Zhang et al., 2021) (Greiling et al., 2018; Chen et al., 2021; Wen et al., 2021; Zhang et al., 2021) (Luo et al., 2018; Wen et al., 2021) (Li et al., 2019; Chen et al., 2021) (Greiling et al., 2018; Azzouz et al., 2019; Wen et al., 2021; Zhang et al., 2021) (Chen et al., 2021; Wen et al., 2021) (Hevia et al., 2014; Greiling et al., 2018; van der Meulen et al., 2019)
MS (9/16)	*Actinomyces* *Akkermansia* (*muciniphila*) *Clostridium* (III, *leptum*) *Eggerthella* (*lenta*)*Streptococcus (anginosus, parasanguinis, salivarius/thermophilus)*	(Cekanaviciute et al., 2017; Forbes et al., 2018) (Jangi et al., 2016; Cekanaviciute et al., 2017; Takewaki et al., 2020) (Forbes et al., 2018; Takewaki et al., 2020) (Miyake et al., 2015; Forbes et al., 2018)	*Bacteroides (coprocola, coprophilus, stercoris)* *Butyricimonas* *Clostridium (sp) Eubacterium rectale* *Faecalibacterium* *Lachnospira (pectinoschiza)* *Lactobacillus* (*rogosae*) *Megamonas funiformis* *Parabacteroides* *Prevotella* (*9, copri*) *Sutterella (wadsworthensis)*	(Miyake et al., 2015; Tremlett et al., 2016) (Jangi et al., 2016; Duscha et al., 2020) (Miyake et al., 2015; Cekanaviciute et al., 2017; Takewaki et al., 2020) (Miyake et al., 2015; Takewaki et al., 2020) (Miyake et al., 2015; Forbes et al., 2018) (Miyake et al., 2015; Tremlett et al., 2016; Forbes et al., 2018) (Miyake et al., 2015; Chen et al., 2016a) (Miyake et al., 2015; Takewaki et al., 2020) (Chen et al., 2016a; Cekanaviciute et al., 2017) (Miyake et al., 2015; Jangi et al., 2016; Cekanaviciute et al., 2017; Zeng et al., 2019) (Miyake et al., 2015; Jangi et al., 2016)
RA (7/5)	*Bacteroides* (*sartorii*) *Eggerthella* *Prevotella* (*amnii, copri, corporis, denticola, disiens, marshii*) *Streptococcus, Streptococcaceae*	(Rodrigues et al., 2019; Sun et al., 2019; Kishikawa et al., 2020) (Chen et al., 2016b; Forbes et al., 2018) (Scher et al., 2013; Maeda et al., 2016; Rodrigues et al., 2019; Kishikawa et al., 2020) (Chen et al., 2016b; Forbes et al., 2018)	*Ruminococcaceae*	(Forbes et al., 2018; Sun et al., 2019)
SS (6/8)	*Prevotella* *Streptococcus* *Veillonella*	(Cano-Ortiz et al., 2020; Mendez et al., 2020) (Bellocchi et al., 2019; Cano-Ortiz et al., 2020) (Cano-Ortiz et al., 2020; Moon et al., 2020a)	*Bifidobacterium* *Blautia* *Dorea* *Faecalibacterium* *Lachnospira* *Firmicutes/Bacteroidetes ratio	(Mandl et al., 2017; Cano-Ortiz et al., 2020; Moon et al., 2020a) (Cano-Ortiz et al., 2020; Moon et al., 2020a) (Cano-Ortiz et al., 2020; Moon et al., 2020a) (Cano-Ortiz et al., 2020; Mendez et al., 2020) (Bellocchi et al., 2019; Cano-Ortiz et al., 2020) (van der Meulen et al., 2019; Cano-Ortiz et al., 2020; Moon et al., 2020a)

The microbiome data presented for each disease are based on the statistical significance of each paper (p < 0.05, q < 0.1, or FDR < 0.1). Parentheses indicate species, but species do not classify all genera. Also, some species are not classified for abundance at the genus level. The microbiome analysis in each paper was performed using human fecal samples. *: No count.

### 2.2 Altered gut bacteria in MS

MS is an autoimmune disease in which the immune system destroys the myelin sheaths surrounding nerve axons in the central nervous system (CNS). MS is on the rise in developed countries and occurs three times higher in young women, for which an environmental factor, such as gut dysbiosis, than genetic factors seems to account (Miyake et al., 2015; Cekanaviciute et al., 2017; Duscha et al., 2020). This assumption is supported by the fact that the transfer of feces from MS patients exacerbates the disease in the animal models of MS (Berer et al., 2017). A total of 16 altered gut bacterial genera were found from the review of nine papers published since 2015 (**Table 1**). While *Actinomyces*, *Akkermansia*, *Clostridium*, *Eggerthella*, and *Streptococcus* are enriched, *Bacteroides*, *Butyricimonas*, *Clostridium*, *Eubacterium*, *Faecalibacterium*, *Lachnospira*, *Lactobacillus*, *Megamonas*, *Parabacteroides*, *Prevotella*, and *Sutterella* are depleted in the gut of MS patients. *Eggerthella* and *Streptococcus* are associated with the induction of autoimmunity (Valour et al., 2014; Ravindra et al., 2018; Cunningham, 2019; Alexander et al., 2022), whereas *Butyricimonas* and *Faecalibacterium* have reported anti-inflammatory functions (Jing et al., 2021; Bai et al., 2022; Houtman et al., 2022), as detailed in Section 2.6. Interestingly, the relative abundance of *Prevotella_9* and *Sutterella* increased in experimental autoimmune encephalomyelitis mice after receiving fecal microbiota transplantation from healthy mice, which, in turn, improved clinical scores (Wang et al., 2021). These results suggest that beneficial bacteria in the host may maintain the homeostasis of the immune system in MS.

### 2.3 Altered gut bacteria in RA

RA is a chronic autoimmune disease that causes joint destruction and functional impairment. Recently, the etiology of RA has been hypothesized to be a combination of genetic factors and gut dysbiosis (Maeda et al., 2016; Jubair et al., 2018). Particularly, the concordance rate for RA in monozygotic twins studied in Europe is 15 percent, which is insufficient to solely explain its etiology by genetic influences (Aho et al., 1986; Silman et al., 1993). Autoantibody production against citrullinated peptides produced by *Porphyromonas gingivalis* is a mechanism to induce RA (Kishikawa et al., 2020). Anti-citrullinated protein antibodies (ACPAs) have been detected in all high-risk RA patients and 93 percent of patients with RA (Tong et al., 2019). A total of five altered gut bacterial genera/families were identified from the review of seven papers published since 2013 (**Table 1**). All four enriched genera, *Bacteroides*, *Eggerthella*, *Prevotella*, and *Streptococcus*, have known functions associated with the induction of inflammatory response or autoimmunity in immune-related diseases detailed in Section 2.6.

### 2.4 Altered gut bacteria in SS

SS is an autoimmune disease characterized by dry mouth and dry eyes (keratoconjunctivitis sicca). Eight genera were identified from the review of six papers published since 2017 (**Table 1**). While *Prevotella*, *Streptococcus*, and *Veillonella* are enriched, *Bifidobacterium, Blautia*, *Dorea*, *Faecalibacterium*, and *Lachnospira* are depleted in the gut bacteriota of SS patients. Among these, *Prevotella* and *Streptococcus* have known functions associated with the induction of inflammatory response or autoimmunity in immune-related diseases, whereas *Bifidobacterium* and *Faecalibacterium* have anti-inflammatory mechanisms of action or efficacy, as detailed in Section 2.6 (Maeda et al., 2016; O’Callaghan and van Sinderen, 2016; Cunningham, 2019; Yao et al., 2021; Houtman et al., 2022).

### 2.5 Altered gut bacteria shared among the four autoimmune diseases versus those unique to each disease

We posed the pertinent question of whether altered gut bacteria are shared among the four autoimmune diseases. The altered taxa listed in **Table 1** were classified into those shared among the four autoimmune diseases (**Table 2**) and those unique to each autoimmune disease (**Table 3**).

**Table 2:** Altered gut bacteria shared in different autoimmune diseases.

Diseases	Enrichment	Depletion
SLE, MS, RA, SS	*Streptococcus*	
SLE, MS, SS	*Streptococcus*	*Faecalibacterium*
SLE, RA, SS,	*Prevotella* *Streptococcus*	
SLE, MS, RA	*Eggerthella* *Streptococcus*	
SLE, MS	*Clostridium* *Eggerthella* *Streptococcus*	*Faecalibacterium*
SLE, SS	*Prevotella* *Streptococcus*	*Faecalibacterium* Firmicutes/Bacteroidetes ratio
SLE, RA	*Bacteroides* *Eggerthella* *Prevotella* *Streptococcus*	*Ruminococcaceae*
MS, SS	*Streptococcus*	*Faecalibacterium* *Lachnospira*

**Table 3:** Altered gut bacteria unique to each autoimmune disease.

Diseases	Enrichment	Depletion
SLE	*Alistipes* *Bacilli* *Escherichia* *Klebsiella* *Lactobacillus*	*Dialister* *Odoribacter* *Roseburia*
MS	*Actinomyces* *Akkermansia*	*Butyricimonas* *Eubacterium* *Lactobacillus* *Megamonas* *Parabacteroides* *Prevotella* *Sutterella*
RA		
SS	*Veillonella*	*Bifidobacterium* *Blautia* *Dorea*

Thereafter, we could identify taxa shared among four, three, and two diseases in various combinations (**Table 2**). Interestingly, *Streptococcus* was enriched in all four diseases. In addition to *Streptococcus*, *Prevotella* was commonly enriched in SLE, RA, and SS, and *Eggerthella* in SLE, MS, and RA. Meanwhile, SLE, MS, and SS shared the depletion of *Faecalibacterium* and the enrichment of *Streptococcus*. Comparing two autoimmune diseases, the SLE–MS, SLE–SS, and SLE–RA combinations shared at least four altered gut bacterial taxa, and the MS–SS combination shared three altered gut bacterial genera. Collectively, SLE shared altered gut bacteria (SAGB) through virtually all comparison groups.

In the case of uniquely altered gut bacteria in each autoimmunity, SLE, MS, and SS had eight, nine, and four genera, respectively, whereas RA had none (**Table 3**). The abundance of *Lactobacillus* was changed in SLE and MS, but in the opposite direction—enriched in SLE but depleted in MS. In addition, the depletion of *Prevotella* was unique to MS.

### 2.6 Potential contribution of altered gut bacteria to disease pathogenesis

To further understand the role of altered gut bacteria in the etiology of autoimmune diseases, we investigated whether the altered bacteria listed in **Table 1** have known functions in immune-related diseases. Studies on gut bacteria’s role and mechanism of action of gut bacteria in human autoimmunity are still limited. Thus, studies on immune-related diseases were also included. We hypothesized that the altered gut bacteria shared among different diseases might be associated with common immunologic pathways of the diseases and that the bacteria unique to each disease may be associated with the specific characteristics of the diseases.

*Streptococcus*, enriched in all four autoimmune diseases, produces antigens that are cross-reactive with host-derived antigens (Cunningham, 2019). These cross-reactive antigens can activate T cells and contribute to autoantibody production through molecular mimicries—hallmarks of autoimmune diseases. This is the third model of immunopathology proposed by Chen et al. for autoimmune mechanisms (Chen et al., 2017). *Streptococcus mutans/sanguinis* bind to salivary proteins and glycoproteins to form biofilms (Matsumoto et al., 2004; Xie et al., 2020). In addition, bacterial biofilms are rich in bacterial extracellular DNA complexed with amyloid, which stimulates autoimmunity (Gallo et al., 2015; Andreasen et al., 2019; Qiu et al., 2019). Thus, DNA abundant in *Streptococcus*-induced biofilms might contribute to autoantibody production by forming a complex with *E. coli*-derived curli amyloid in the gut environment (Chen et al., 2019; Qiu et al., 2019; Barrasso et al., 2022). These results suggest that *Streptococcus* may be closely related to the development of autoimmune diseases through autoantibody production. However, further understanding of *Streptococcus* species and their strains involved in disease etiology is needed.

*Eggerthella lenta* is commonly enriched in SLE, MS, and RA (**Tables 1**, **2**). In an inflammatory bowel disease (IBD) model, *E. lenta* activates Th17 cells through the cardiac glycoside reductase 2 (Cgr2) enzyme, which metabolizes endogenous Rorγt inhibitors (Alexander et al., 2022). However, the activation of Th17 cells by *E. lenta* is affected by two variables. First, a high concentration of dietary arginine (3 percent/kg) can inhibit the function of the Cgr2 enzyme (Alexander et al., 2022). Second, *E. lenta* does not express Cgr2 depending on the strain, and Cgr2^-^ strains do not activate Th17 cells. This result indicates that the contribution of *E. lenta* to the development of autoimmune diseases may depend on host dietary factors and bacterial strains. This finding relates to the first immunopathology model proposed by Chen et al. for autoimmune mechanisms (Chen et al., 2017). In addition, *E. lenta* was enriched in the gut of mice exposed to cigarette smoke for seven months (Bai et al., 2022). Furthermore, the transplantation of feces from smoking-exposed mice into germ-free mice induced enrichment of *E. lenta*, an impairment of the gut barrier in the colonic epithelium, and an increase in proinflammatory cytokines IL-17 and TNF (Bai et al., 2022). Notably, smoking is a common risk factor for SLE, MS, and RA (Majka and Holers, 2006; Healy et al., 2009; Amador-Patarroyo et al., 2012; Mowry et al., 2012; Baka et al., 2009), and Th17 cells are involved in the pathogenesis of these three diseases (Yang et al., 2019; Moser et al., 2020; Robert and Miossec, 2020).

*Prevotella* is enriched in SLE, RA, and SS but depleted in MS. Specifically, *P. copri* is enriched in RA but depleted in MS (**Table 1**). Interestingly, the colonization of germ-free mice with fecal samples from RA patients dominated by *P. copri* induced a Th17 cell-dependent autoimmune arthritis, suggesting that gut dysbiosis with enriched *P. copri* contributes to the development of RA (Maeda et al., 2016). Kishikawa et al. also suggested that enriched multiple *Prevotella* spp. are associated with the etiology of RA in Japanese patients (Kishikawa et al., 2020). However, clinical trials of IL-17 blockers presented limited clinical efficacy in RA compared with their efficacies in psoriasis or psoriatic arthritis (Schett et al., 2013; Fauny et al., 2020). This suggests that the roles of Th17 cells and IL-17 in the etiology of RA may be multi-faceted, as the presence of Foxp3^+^IL-17^+^ T cells is observed in the subjects’ synovium (Komatsu et al., 2014; Dominguez-Villar and Hafler, 2018). Multiple *Prevotella* spp. have been suggested to be associated with the etiology of RA (**Table 1**); however, their mechanisms of action are more complex than previously recognized. Therefore, the roles of Th17 subtypes and multiple *Prevotella* spp. in the etiology of RA need to be clarified (Omenetti et al., 2019). Considering the role of Th17 cells in the pathogenesis of MS, further investigation is needed to determine the role of *P. copri* depletion in MS etiology.

*Faecalibacterium* is commonly depleted in SLE, MS, and SS (**Tables 1**, **2**). *Faecalibacterium* maintains homeostasis of the gut immune system by secreting anti-inflammatory compounds such as (Houtman et al., 2022), salicylic acid (Miquel et al., 2015), and microbial anti-inflammatory molecules (Quevrain et al., 2016). In addition, *F. prausnitzii* and its supernatant effectively increase the function of Short-chain fatty acid (SCFA)-producing bacteria (Zhou et al., 2021). SCFAs are produced through the breakdown of various indigestible dietary fibers and complex carbohydrates catalyzed by the gut microbiota (Park and Kim, 2021). Beneficial bacteria in the oral cavity and gut of healthy individuals can modulate the inflammatory response through the secretion of immunomodulators such as SCFAs (acetate, butyrate, and propionate) (Feng et al., 2018; Dalile et al., 2019; Mendez et al., 2020; Vijay and Valdes, 2021; Houtman et al., 2022). In addition, *Faecalibacterium*, which secretes SCFAs such as butyrate, is well known for its anti-inflammatory properties (Van de Wiele et al., 2016).

The anti-inflammatory effect of SCFAs is mediated through the induction of Treg cells and the alleviation of disease symptoms (Machiels et al., 2014; Kim et al., 2017; Moon et al., 2020b; Vijay and Valdes, 2021). Specifically, among the three types of SCFAs, butyrate and propionate were effective in inducing Foxp3, but acetate was not [untreated 30.4 percent, acetate 31.4 percent, propionate 41.9 percent (p < 0.01), and butyrate 54.2 percent (p < 0.01)] (Furusawa et al., 2013). In patients with relapsing-remitting MS (RRMS), SCFA concentrations in the fecal samples were significantly reduced compared to controls (Takewaki et al., 2020). However, the hypersecretion of SCFAs may also lead to side effects, such as bacterial invasion associated with the reduced mucus layer and inflammation (Gaudier et al., 2009; Park et al., 2016; Clarke, 2020; Okumura et al., 2021). Butyrate enemas reduced the thickness of the adherent mucus layer by approximately two-fold when administered to mice (Gaudier et al., 2009). The fact that RA developed only in mice with increased gut permeability suggests that bacterial invasion may be associated with a decrease in the mucus layer (Clarke, 2020). These results suggest that the decrease and hypersecretion of SCFAs may be related to the etiology of autoimmune diseases, which are long-term chronic diseases. Thus, more detailed studies on the role of SCFAs in autoimmune diseases may be needed.

*Bacteroides* are enriched in SLE and RA (**Table 1**). The structure and function of *Bacteroides*-derived LPS have been shown in relation to the development of type 1 diabetes (T1D). The immunostimulatory efficacy of *Bacteroides*-derived LPS was four times lower than that of *Escherichia coli*-derived LPS. While the *E. coli*-derived LPS delayed the onset of T1D in non-obese diabetic mice by inducing endotoxin resistance, *Bacteroides*-derived LPS neither induced endotoxin resistance nor delayed the development of T1D (Vatanen et al., 2016). As a result, *Bacteroides*-derived LPS caused more inflammatory responses than *E. coli*. A similar mechanism may play a role in the pathogenesis of SLE and RA. However, SLE patients also have depleted species that belong to the *Bacteroides* genus. In this context, species-specific modulation of immune function by *Bacteroides* must be studied.

*E. coli*, enriched in SLE, can be divided into pathogenic and nonpathogenic strains (Palmela et al., 2018; Lorenz et al., 2020). Infection with *E. coli* expressing curli amyloid can induce the production of autoantibodies by forming a complex with DNA derived from bacteria or viruses. The amyloid/DNA complexes produce anti-nuclear autoantibodies and anti-dsDNA autoantibodies involved in SLE pathogenesis (Dema and Charles, 2016; Qiu et al., 2019). This was verified because curli amyloid-deficient mutant *E. coli* does not produce autoantibodies (Gallo et al., 2015). This finding may be related to the fifth model of immunopathology proposed by Chen et al. for autoimmune mechanisms (Chen et al., 2017).

Although *Ruminococcus gnavus* is a gram-positive bacterium, the complex glucorhamnan polysaccharide secreted from this bacterium induces TNFα through TLR4 in dendritic cells (Henke et al., 2019). In contrast, *Ruminococcus_2* is associated with the improvement of metabolic dysfunction. For example, the consumption of barley for eight months in subjects with metabolic dysfunction improved blood sugar levels and cholesterol levels, which accompanied the enrichment of *Ruminococcus_2* and *Dialister* in the subjects’ gut (Matsuoka et al., 2022). This suggests that the depletion of these commensal bacteria may be associated with the development of metabolic dysfunction. Abnormal metabolic reactions, such as elevations in glycolysis and mitochondrial oxidative metabolism, have also been reported in patients with SLE (Yin et al., 2015; He et al., 2020). Gut dysbiosis in patients with SLE includes enrichment of *R. gnavus* and depletion of *Ruminococcus_2* and *Dialister* (**Table 1**). These results suggest that abnormal metabolism in SLE may be closely associated with gut dysbiosis.

A mouse model of spinal cord injury shows the neuroprotective effects of *Butyricimonas*, a genus depleted in patients with MS. *Butyricimonas* is depleted in mice with spinal cord injury but recovers by fecal microbiome transfer from healthy mice, which induces downregulated IL-1β and NF-κB signaling in the spinal cord (Jing et al., 2021). Therefore, these results suggest that the depletion of *Butyricimonas* in patients with MS may be closely related to its etiology (**Table 1**).

The *Bifidobacterium* genus was reported to be depleted in patients with SS in three papers (**Table 1**) (Mandl et al., 2017; Cano-Ortiz et al., 2020; Moon et al., 2020a). However, this commensal bacterium needed to be further classified for comparative analysis with other diseases because its relative abundance in gut dysbiosis differed depending on the species. For example, *B. longum* is effective in preventing IBD and treating diarrhea (O’Callaghan and van Sinderen, 2016; Yao et al., 2021). On the other hand, *B. bifidum* can induce the differentiation of Th17 cells (Rinaldi et al., 2019). Based on these results, the *Bifidobacterium* genus in SS, an autoimmune disease, is likely to be *B. longum*, but it remains a task to be identified at the species level in the future.

We also investigated how many targeted therapies are shared among the four autoimmune diseases. This is because the altered gut bacteria that may be associated with the etiology of the disease are shared in autoimmune diseases. Petitdemange et al. reported targeted therapies shared in autoimmune or inflammatory diseases (Petitdemange et al., 2020). Four targeted therapies (abatacept, anakinra, ianalumab, and rituximab) are shared among the four autoimmune diseases (**Table 4**). Among the targeted therapies shared by three diseases, seven (alemtuzumab, atacicept, evobrutinib, ocrelizumab, secukinumab, tabalumab, and ustekinumab) are shared among SLE, MS, and RA. Furthermore, seven (belimumab, etanercept, filgotinib, iscalimab, lanraplenib, omalizumab, and telitacicept) are shared among SLE, RA, and SS, and one (baminercept) is shared among MS, RA, and SS. These results suggest that targeted therapies in autoimmune diseases are related to overlapping immunological pathways due to common causes. It is tempting to say that the altered gut bacteria shared among diseases might be partially involved (Petitdemange et al., 2020). In particular, 52.6 percent (10 out of 19) of the treatments for these four diseases consisted of molecules that target B cells or antibody production, indicating that the altered gut bacteria may be closely related to autoantibody production.

**Table 4:** Targeted therapies and commensal bacteria shared by four autoimmune diseases.

Diseases	Targeted therapies (TGT)	Mechanism of action of TGT	Shared altered gut bacteria (SAGB)	Mechanism of action of SAGB
SLE, MS, RA, SS	Abatacept Anakinra Ianalumab Rituximab	CTLA4-Ig fusion protein IL-1R antagonist Anti-BAFF receptor mAb Anti-CD20 mAb	*Streptococcus* *Streptococcus*	Autoantibody production Autoantibody production
SLE, MS, RA	Alemtuzumab Atacicept Evobrutinib Ocrelizumab Tabalumab Secukinumab Ustekinumab	Anti-CD52 mAb BAFF and APRIL inhibitor BTK (B cell development) inhibitor Anti-CD20 mAb Anti-BAFF mAb Anti-IL-17 mAb Anti-IL-12 and IL-23 mAb	*Streptococcus* *Streptococcus* *Streptococcus* *Streptococcus* *Eggerthella lenta*, *Prevotella* spp.	Autoantibody production Autoantibody production Autoantibody production Autoantibody production IL-17 production
SLE, RA, SS	Belimumab Telitacicept Etanercept Filgotinib Iscalimab Lanraplenib Omalizumab	Anti-BAFF mAb BAFF and APRIL inhibitor TNFα inhibitor JAK1 inhibitor Anti-CD40 mAb SYK-kinase inhibitor Anti-IgE mAb	*Streptococcus* *Streptococcus* *Streptococcus* *Streptococcus*	Autoantibody production Autoantibody production Autoantibody production Autoantibody production
MS, RA, SS	Baminercept	LT beta receptor-Ig fusion protein		

mAb, monoclonal antibody; LT, Lymphotoxin.

### 2.7 Association between shared gut bacteria and polyautoimmunity

Polyautoimmunity can be defined as the coexistence of one or more autoimmune diseases in one patient (Rojas-Villarraga et al., 2012). Polyautoimmunity in patients with SLE, SS, and RA has a prevalence of 41 percent, 32.6 percent, and 14 percent, respectively (Ordonez-Canizares et al., 2022). Although data on overall polyautoimmunity in patients with MS are unavailable, the prevalence of coexisting SS has been suggested to be between 1 and 16.6 percent (Amador-Patarroyo et al., 2012). These results may be related to the fact that autoimmune diseases share altered gut bacteria associated with the failure to maintain immune homeostasis (**Table 2**). The enriched relative abundance of *Bacteroides*, *Eggerthella*, *Prevotella*, and *Streptococcus*, shared in autoimmune diseases, has been reported to be related to the promotion of immune responses in immune-related diseases. This is due to Bacteroides-derived LPS, metabolizing Rorγt inhibitors, Th17 cell induction, and antibodies to cross-reactive antigens, respectively (Maeda et al., 2016; Vatanen et al., 2016; Cunningham, 2019; Alexander et al., 2022). In particular, as aforementioned, *Streptococcus*, which is shared by all four autoimmune diseases, has been suggested to be involved in autoantibody formation (Cunningham, 2019). This result might also be partially related to the fact that many therapies for these four diseases involve the inhibition of autoantibody production (**Table 4**) (Petitdemange et al., 2020).

Meanwhile, SLE, MS, and SS patients showed a decreased abundance of *Faecalibacterium* abundance. The decrease of *Faecalibacterium* has the potential to significantly impact the etiology of autoimmune diseases because they secrete various immune modulators, such as butyrate, salicylic acid, and microbial anti-inflammatory molecules, as aforementioned (Miquel et al., 2015; Quevrain et al., 2016; Houtman et al., 2022). These results suggest that the altered gut bacteria shared between autoimmune diseases might contribute to the development of polyautoimmunity (De Luca and Shoenfeld, 2019; Xu et al., 2021). However, a direct causal relationship between the shared, altered gut bacteria and polyautoimmunity remains to be further elucidated.

### 2.8 Association between altered gut bacteria and mortality

**Figure 1:**
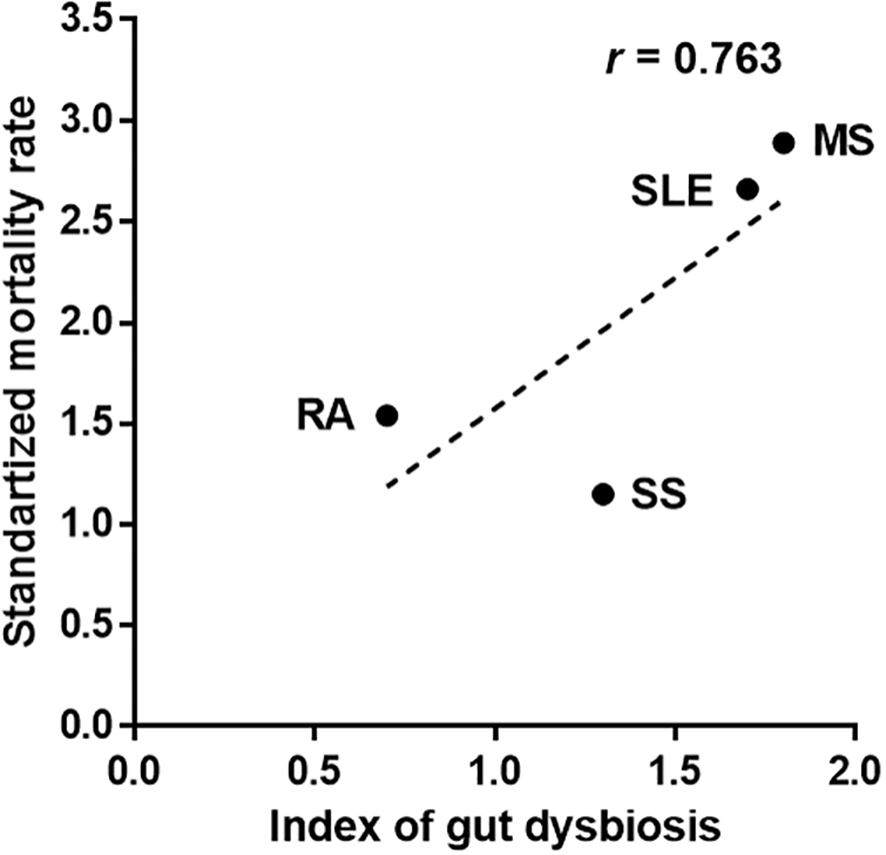
Association between the number of altered gut bacteria and mortality in patients with autoimmune diseases. The index of gut dysbiosis is defined as the number of altered gut bacterial taxa divided by the number of studies. R is Pearson’s correlation coefficient.

The total number of altered gut bacteria in each autoimmune disease differed depending on the disease—22 in SLE, 16 in MS, 5 in RA, and 8 in SS (**Table 1**). As the number of altered taxa cross-validated across different studies can increase with the increased number of studies, we defined a gut dysbiosis index as the number of altered gut bacterial taxa divided by the number of studies. The gut dysbiosis indexes of SLE, MS, RA, and SA were 1.7, 1.8, 0.7, and 1.3, respectively. Based on these results, we investigated whether a higher degree of gut dysbiosis in SLE and MS was associated with mortality. The most recent papers from developed countries were used for similar comparative conditions for mortality due to each disease. The SMR of patients with MS was 2.89 [95 percent CI, 2.71 to 3.07; UK (period: 1980–2007)], indicating a 189 percent higher risk of death than the general population (Kingwell et al., 2012). The SMR of patients with SLE was 2.66 [95 percent CI, 2.09 to 3.39; Korea (period: 1990–2015)], indicating a 166 percent higher risk of death than the general population (Lee et al., 2016). However, the SMRs of patients with RA and SS were 1.54 [95 percent CI, 1.41 to 1.67; Netherlands (period: 1997–2012)] (van den Hoek et al., 2017) and 1.15 [95 percent CI, 0.86 to 1.50; USA (period: 2006–2015)] (Maciel et al., 2017), respectively. These values were slightly higher than or no different from the general population. Interestingly, the SMRs presented a positive correlation trend with the gut dysbiosis indexes (**Figure 1**), suggesting that a high degree of gut dysbiosis may adversely affect immune homeostasis and increase mortality rates.

## 3 Conclusion

The importance of gut dysbiosis in the etiology of autoimmune diseases is increasing. Thus, to better understand the impact of gut dysbiosis, we first investigated the cross-validated altered gut bacteria in each disease and further analyzed the altered gut bacteria shared between autoimmune diseases. Interestingly, the shared, altered gut bacteria enriched in autoimmune diseases are partially related to autoantibody production or the activation of Th17 cells in reports of immune-related diseases. In particular, the decrease of *Faecalibacterium* shared in SLE, MS, and SS, which secretes various immunomodulatory substances, can greatly affect the failure to maintain immune homeostasis. The SMR in patients with SLE and MS was higher than that of RA and SS, which was shown to be positively correlated with the total number of altered gut bacteria in four autoimmune diseases. In taxonomic abundance analysis, *Bifidobacterium*, *Bacteroides*, *Lactobacillus*, *Prevotella*, and *Ruminococcus* should be classified at the species level, not the genus level, as their relative abundance may vary depending on the species. However, the abundance of *Bacteroides fragilis* and *Prevotella copri* varies according to diseases or strains, even if the species are the same, so further research is needed. In addition, the non-cross-validated microbiome, excluded from this study, is left for future tasks by accumulating more data. This review suggests that the altered gut bacteria in patients with autoimmune diseases may be closely related to abnormal immune activity and weakened anti-inflammatory activity. In addition, the increased number and sharing of altered gut bacteria are likely to be associated with disease exacerbation and polyautoimmunity, respectively. However, the direct causal relationship between altered gut bacteria and each autoimmune disease remains to be clarified.

## Author contributions

S-HC and YC contributed the concept and design of the paper. S-HC and YC participated in the review and editing. All authors contributed to the article and approved the submitted version.
